# Cardiovascular System Involvement in Cystic Fibrosis

**DOI:** 10.7759/cureus.16723

**Published:** 2021-07-29

**Authors:** Prutha H Shah, Jun Hee Lee, Dhairya J Salvi, Rizwan Rabbani, Divya R Gavini, Pousette Hamid

**Affiliations:** 1 Internal Medicine, Pediatrics, California Institute of Behavioral Neurosciences & Psychology (CIBNP), Fairfield, USA; 2 Internal Medicine, California Institute of Behavioral Neurosciences & Psychology (CIBNP), Fairfield, USA; 3 Nephrology, California Institute of Behavioral Neurosciences & Psychology (CIBNP), Fairfield, USA; 4 Neurology, California Institute of Behavioral Neurosciences & Psychology (CIBNP), Fairfield, USA

**Keywords:** cystic fibrosis, cardiovascular system, cftr, myocardial fibrosis, cor pulmonale

## Abstract

Cystic fibrosis (CF) is an autosomal recessive disease primarily affecting the respiratory system and gastrointestinal system. The life expectancy of patients with CF has significantly improved due to medical advancement and the effective use of screening techniques. However, new challenges have emerged. Particularly those involving cardiovascular pathology. This study aims to provide a better understanding of the different mechanisms that cause cardiovascular complications in patients with CF, which would help find an efficient treatment that not only prolongs survival but also improves their quality of life. This study extensively reviews different theories such as right ventricular hypertrophy due to lung pathology, ventricular interdependence, the association of nutritional deficiencies and severe cystic fibrosis transmembrane conductance regulator (CFTR) genotypes with myocardial fibrosis, effects of hypoxia, recurrent infections, and systemic inflammation of the heart and blood vessels that explain the direct or indirect involvement of the cardiovascular system in CF. For this review, 258 articles were retrieved from PubMed and Google Scholar. Out of which, a total of 12 high-quality articles were selected using appropriate quality assessment tools and preferred reporting items for systematic reviews and meta-analyses (PRISMA) guidelines. The result of this study suggests that early detection of cardiovascular dysfunction can improve the survival rate of the patient. Furthermore, this study could aid future researchers in the exploration of various best screening modality techniques for the early detection of cardiovascular dysfunction.

## Introduction and background

Cystic fibrosis (CF) is the most prevalent fatal inherited disease among Caucasians [1]. It is caused by defects in the CF gene located on the seventh chromosome, which encodes for the cystic fibrosis transmembrane conductance regulator (CFTR). CFTR operates a chloride ion (Cl-) channel that is regulated by cyclic adenosine monophosphate (cAMP). A defect in CFTR results in reduced Cl-^ ^secretion and increased reabsorption of sodium ion (Na+) and water across the epithelium. Subsequently, decreased epithelial-lining fluid and reduced hydration of mucus result in mucus that is more sticky to bacteria thereby leading to recurrent infection and inflammation. The increased viscosity of secretions in the respiratory tract, sweat glands, gastrointestinal tract, pancreas, and other exocrine tissues, makes these secretions difficult to clear. This leads to recurrent respiratory infections, insufficient pancreatic enzymes, and accompanying complications in patients with CF. Involvement of the lungs is seen in 90% of patients who survived the neonatal period. The major cause of death in CF is end-stage lung disease.

According to the Cystic Fibrosis Foundation Patient Registry (CFFPR), the United States has over 30,000 people living with CF (> 70,000 worldwide) with roughly 1,000 new cases of CF diagnosed every year [2].

This disease primarily affects the respiratory system and the gastrointestinal system. However, recent studies suggest a direct or indirect involvement of the cardiovascular system. The destruction of lung parenchyma and pulmonary vasoconstriction due to chronic hypoxemia may result in pulmonary hypertension causing cor pulmonale. Some studies imply that the CFTR protein is also expressed in cardiac myocytes, including both atrial and ventricular myocytes [3] and blood vessels [4]. Just like epithelial CFTR, cardiac CFTR also conducts Cl-, and loss of its function influences myocyte contractility, intracellular calcium signaling, myocardial fibrosis, and heart remodeling [5,6].

With the advancement of medical science and effective screening tests, the life expectancy of patients with CF has improved dramatically in the past few decades. Hence, the number of middle-aged patients with CF has increased. Betterment of health has certainly brought hope to these patients. On the other hand, it has given rise to new challenges. This study mainly focuses on one of those potential challenges, namely cardiovascular system involvement in patients with CF. The study also identifies the potential mechanisms causing this association and assesses its relation to the severity of the disease. A better understanding of the cardiac function in CF might help improve the quality as well as longevity of life.

## Review

Methodology

The Study-Protocol

In this review, the preferred reporting items for systematic and meta-analyses (PRISMA) guidelines [7] were used.

The Source of Data Collection

To write this paper, two databases were used: Google Scholar and PubMed. A multi-dimensional, systematic search was carried out on the data published in the last 25 years i.e., 1996 to 2021, and was surveyed to identify relevant articles. For the data collection in PubMed, medical subject headings** (**MeSH) along with regular keywords were used. As for Google Scholar, only regular keywords were used.

Inclusion-Exclusion Criteria

There were five inclusion criteria implemented for data collection: One, to include studies carried out over the last 25 years with a focus on newer studies. Two, to incorporate studies that were conducted solely on patients with CF. Three, to feature studies carried out on humans and animals. Four, choose studies written only in English. And five, identify studies done on children and adults with CF. As for the exclusion criteria, systematic reviews/review articles were excluded.

Search Content

To identify the articles, the following keywords were used. 

Regular keywords: Cystic fibrosis, heart, cardiovascular system, cor pulmonale

Pulmonale MeSH keywords: ("cystic fibrosis/complications"[MeSH]) AND ("pulmonary heart disease"[MeSH]) OR ("cystic fibrosis/complications"[MeSH]) AND (heart/abnormalities"[MeSH]) OR ("heart/adverse effects"[MeSH]) OR ("heart/complications"[MeSH]) OR ("heart/etiology"[MeSH]) OR ("cystic fibrosis/complications"[MeSH]) AND ("heart"[MeSH]) OR ("cystic fibrosis/complications"[MeSH]) AND ("heart ventricles"[MeSH]) OR ("cystic fibrosis/complications"[MeSH]) AND ("echocardiography"[MeSH])

The content of all the articles was reviewed and only relevant articles were selected.

Ethical Issue

The goal of this paper is to systematically review all of the published data. To do so, the studies were collected in a systematic, accurate, and adequate manner.

Quality Check

The quality of included articles has been evaluated by employing the Newcastle Ottawa scale for case-control and cohort studies. Also, nine qualitative assessment questions compiled by the National Institutes of Health (NIH) were posed at these studies, and the answers to them were used to determine the case series. Low-quality papers have been excluded and only moderate and high-quality papers included.

Data Search

**Figure 1:**
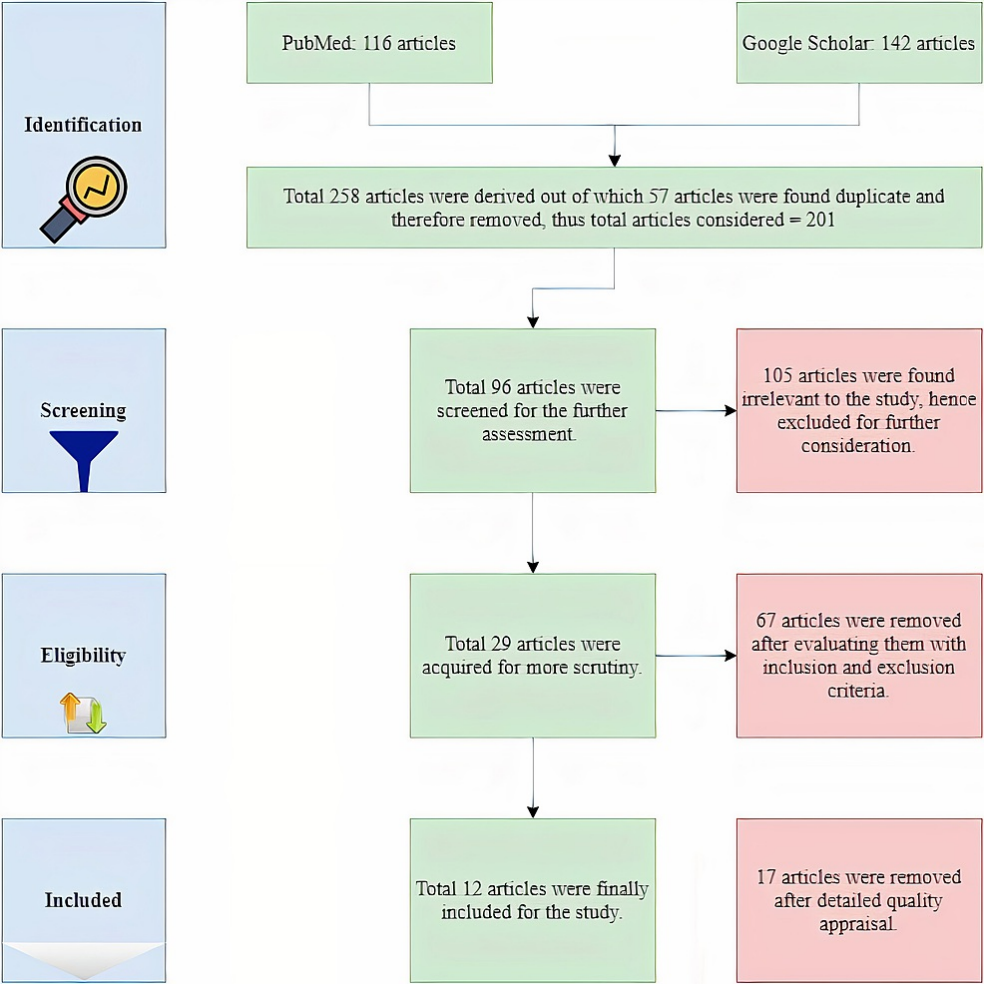
PRISMA flow diagram

With the help of regular and MeSH keywords, a total of 258 articles were found. A predominant number of the articles, 142 to be precise, were from Google Scholar, and the remaining 116 were from PubMed. Fifty-seven of these articles were excluded as they were duplicates. A further 105 articles were excluded as they were found not to be relevant to the study. A total of 96 full-text articles were screened for further assessment. Sixty-seven of these articles were removed after evaluating the inclusion-exclusion criteria, and 29 articles were acquired for further scrutiny. After using appropriate tools for quality assessment, another 17 articles were removed. And finally, 12 articles were rounded up for the study. This process to identify relevant articles for the study is represented below in Figure 1.

Discussion

Genetics

Cystic fibrosis (CF), is an autosomal recessive disease that is caused by mutations in the CFTR gene. The presence of non-functional CFTR on the apical membrane of secretory epithelium results in defective secretion of Cl- and bicarbonate (HCO3), along with increased Na+ absorption and mucus secretion, which causes dehydration and acidification of the airway surface liquid. Consequently, it leads to impaired mucociliary clearance that provokes recurrent pulmonary infections and uncontrolled inflammation causing damage to the lung parenchyma. To date, over 1000 mutations of the CFTR gene that causes cystic fibrosis have been discovered [8].

CFTR protein channel is also found in the cardiovascular system. The existence of CFTR within cardiac myocytes has been known for more than 20 years [9]. An inherent defect in the CFTR protein channel causes ventricular dysfunction. In murine [10], CFTR dysfunction in cardiomyocytes results in an increased concentration of intracellular calcium ions (Ca2+). The loss of CFTR ion transport is compensated by increased activity of calmodulin-dependent protein kinase (CAM kinase) II and calcium-activated chloride channels [5]. Upregulation of the CAM kinase II activity can cause hypertrophy of the left ventricle (LV) and subsequent development of dilated cardiomyopathy [11,12]. Evidence has shown that cystic fibrosis mice have developed heart failure earlier compared to their wild-type controls. Decrease in cardiac reserve on dobutamine stimulation as well as hypertrophy of LV and hyperdynamic hearts [4] are observed on Doppler echocardiography and cardiac catheterization of the heart in deltaF508 (ΔF508) CFTR murine. Another experiment done on the cardiomyocytes of neonatal mice [13,14] has revealed that CFTR is also involved in regulating cardiomyocyte contraction rate. Recent studies done on the ΔF508 mutant mice have detected alterations in cardiac and vascular function in the absence of lung pathology [4]. Loss of CFTR function in them leads to LV remodeling and increased aortic stiffness. This evidence proposes a direct effect of CFTR on cardiac changes [15].

When similar kinds of experiments were conducted on the human heart, the results were inconclusive. The mammalian heart has numerous different Cl- channels in the sarcolemma of ventricular myocytes. One of these molecules is quite similar to the CFTR Cl- channel of epithelium in all the biophysical, biochemical, and pharmacological properties observed to date. Molecular studies have suggested that this may be an isoform of a CFTR protein [16]. However, the role of dysfunctional CFTR in the pathogenesis of myocardial complications in patients with CF seems unjustified as recent evidence suggests that the quantity of CFTR ion channel expressed in human myocardial tissue (atrial and ventricular) might be physiologically inadequate to activate obvious cyclic adenosine monophosphate (cAMP)-dependent Cl- conductance [17,18].

Although more studies are required to completely understand the molecular and cellular changes that occur in the myocardium due to CF and its physiologic consequences, the studies that have been made so far give insight into the possible mechanisms of cardiac dysfunction in CF [19].

Pathophysiology

Left ventricle: Echocardiographic assessment of patients with CF demonstrates that cardiac changes in systolic and diastolic functions can start early in life [20]. Involvement of the right ventricle (RV) has been consistently seen in patients with CF. However, evidence of cardiomyopathy, ventricular arrhythmia, sudden cardiac death, and fibrosis, and necrosis of LV myocardium also indicate LV pathology in cystic fibrosis [19].

Classical methods of assessing LV systolic function, such as left ventricular ejection fraction (LVEF) and fractional shortening, are relatively insensitive. Therefore, these methods are not capable of detecting early sub-clinical changes. Strain and strain rate (SR) was approved as the screening tool [21,22] for identifying sub-clinical cardiomyopathy that causes LV systolic dysfunction [23]. On the other hand, preserved LV systolic function and morphology, and impaired LV diastolic filling has also been detected in patients with CF. These patients are more dependent on the atria for LV filling compared to normal controls or patients with bronchiectasis [24].

Several possible mechanisms can account for functional changes in LV related to cystic fibrosis. For instance, the enlargement of the RV can decrease LV distensibility through the interventricular septum. This mechanism is supported by evidence of septal flattening on echocardiographic observation in some patients with CF [24]. LVEF accessed by two dimensional (2D) echo showed that LV involvement appears to occur when RV is massively enlarged [25]. Other factors responsible for ventricular distensibility are raised levels of circulating neurohormonal mediators and tachycardia that could have a greater influence on the ventricular filling. This may conceal the effect of the RV volume and pressure overload on the LV function [24]. Also, myocardial fibrosis can increase the thickness of the interventricular septum resulting in impaired LV relaxation during diastole [20].

The observation of LV function in patients with CF indicates that the pathologic process in CF may directly affect the myocardium. Numerous case reports have shown myocardial fibrosis in the autopsy of children diagnosed with CF had signs/symptoms of LV dysfunction [24]. So far, myocardial fibrosis and necrosis in patients with CF have been explained by two hypotheses [25]. The first one states that myocardial cytolysis would result from activation of the kinin system via activated pancreatic enzymes such as kallikrein and trypsin that are released into circulation from a diseased pancreas. The second theory explains that myocardial necrosis can be caused by the deficit of vitamins E, B1, A, and microelements among other substances that are essential for maintaining the myocardium. Along with these theories, there is evidence suggesting that genetic factors play a vital role in the development of myocardial lesions. Severe CFTR genotypes, such as ΔF508: ΔF508 and asparagine-to-lysine mutation at position 1303 (N1303K) mutations may be associated with the development of myocardial fibrosis and necrosis [20]. Thus, myocardial fibrosis may result from the co-occurrence of genetic susceptibility to myocardial lesions (severe CFTR genotypes along with the presence of certain modifying genes) and a deficiency of some trophic factors essential for myocardial metabolism [20]. In addition, increased levels of neurohormonal mediators - aldosterone and angiotensin II - might be present in patients with CF [24]. They stimulate the proliferation of fibroblastic cells and the synthesis of protein in myocytes [26] causing myocardial fibrosis [27-29]. This theory might have some therapeutic consequences, such as the angiotensin-converting enzyme (ACE) inhibitors and angiotensin receptor blockers (ARBs) being used in the treatment of patients with CF [23].

Newer studies have revealed that early lung disease may be present among asymptomatic infants with CF who display normal lung function [30]. Hence, we cannot eliminate the theory suggesting that pulmonary disease may play a significant role in the development of cardiovascular lesions in patients with CF. Many of these patients have experienced recurrent pulmonary infections in early life, which leads to the gradual destruction of the lung parenchyma. A higher circulatory level of pro-inflammatory mediators is also present due to recurrent lung infections [15]. These mediators are associated with negative inotropic effects, metabolic disturbance in myocyte, hypertrophy of myocyte, and alterations in the extracellular matrix. These changes have a deleterious effect on the LV function [31]. The relation of inflammatory markers with left ventricular dysfunction is not yet known but warrants more prospective studies [23].

Diabetic cardiomyopathy is implicated as another potential mechanism for LV dysfunction [32]. Constant hyperglycemia is responsible for interstitial protein glycation that results in impaired contractility and increased myocardial stiffness, known as autonomous cardiomyopathy, also seen in diabetic patients [23]. Chronic hypoxia can also be invoked as a potential mechanism for subclinical LV (causes myocardial fibrosis) in patients with CF [23]. Subclinical ventricular dysfunction can also develop as a consequence of pulmonary hypertension [15]. It has been reported that myocardial edema and lymph stasis were seen in two patients who died due to CF-related cardiomyopathy, which suggests that CF can be complicated by impaired lymphatic drainage of the heart [27]. The increased arterial stiffness increases left ventricular afterload and decrease the perfusion of the coronary arteries in the diastole. But the increased arterial stiffness in children with CF is quite subtle. Thus, it is not clear that this increase can fully explain the cardiac changes [15].

Right ventricle: Evidence of cardiac involvement in CF was reported for the first time in 1946. The report stated that hypertrophy of the RV is caused by pulmonary disease [33]. Since then, the structural and functional anomalies of RV have been evident among patients with CF, and these occur primarily due to long-standing hypoxia, progressive chronic obstructive disease, and raised pulmonary pressures [19]. Some of the evidence has shown that right ventricular systolic and diastolic dysfunction may be present in the absence of significant pulmonary disease. These abnormalities are subclinical and not associated with genotypes, pulmonary function test (PFT), clinical scores, pancreatic status, or chronic colonization [34].

Subclinical involvement of the right heart is evidenced by significantly higher systolic pulmonary artery pressure (sPAP), even though these patients do not have pulmonary hypertension, as well as RV diastolic dysfunction (40-60%) [33].

The involvement of RV in patients with CF without obvious pulmonary hypertension has been explained by two hypotheses. First, hypoxemia, systemic inflammation with oxidative stress, and subsequent endothelial dysfunction may affect ventricular contractility and relaxant properties [35, 36]. Patients with CF have relatively higher heart rates and diastolic blood pressure (BP). Higher BP can be the result of oxidative stress on the arterial wall due to chronic inflammation and endothelial dysfunction. Consequently, heart rate will also be increased in parallel with myocardial work. Second, sub-clinical, temporary, and recurring pulmonary hypertension due to the advancement of lung pathology can gradually damage RV properties even in well-controlled patients [37]. It is conceivable that both mechanisms exist together and potentiate one another [33].

Endothelial dysfunction, also known as 'vascular remodeling', is believed to present before the structural changes and it occurs via an imbalance between the production of vasodilative and vasoconstrictive factors [38]. This endothelial dysfunction translates into increased pulmonary vascular resistance and eventually irreversible pulmonary hypertension [25]. Hypoxia, as well as chronic inflammation, causes some degree of remodeling in pulmonary vessels. Hence, pulmonary systolic artery pressure (PSAP) is significantly higher in patients with CF [23] and could ultimately lead to overt changes in RV function [25].

The systolic function of RV, which is measured with tricuspid annular plane systolic excursion (TAPSE) and tissue Doppler imaging, is significantly lower in children with CF [15]. RV systolic dysfunction is caused by the destruction of pulmonary parenchyma, which leads to hypoxia that in turn leads to hypoxic vasoconstriction of the pulmonary vasculature. This is responsible for the increase in pulmonary vascular resistance that in turn results in an increasing afterload. To support this, a correlation between forced expiratory volume during the first second of forced breath (FEV1) and right ventricular ejection fraction (RVEF) is also found [24]. Long-standing pulmonary hypertension results in cor pulmonale (RV enlargement and dysfunction) that might progress to right heart failure (RHF). There is a high occurrence of right ventricular hypertrophy and dilatation in patients with CF. RV dimension is considered to reflect the severity of cor pulmonale in these patients [34]. A strong association between RV systolic dysfunction, measured by the amplitude of long-axis excursion at the RV free wall, and the degree of hypercapnia and hypoxia, expressed by the partial pressure of carbon dioxide (Paco2) and partial pressure of oxygen (Pao2), is also found [39]. Alterations in RV systolic function is more likely a result of the combination of increased pulmonary vascular resistance and long-standing hypoxia [39].

Another possible mechanism is the direct involvement of the heart in patients with CF. Lately, it has been established that CFTR is also involved in regulating cardiomyocyte contraction, and the loss of CFTR function may cause cardiac dysfunction in these patients [25].

Blood vessels: Microvascular function measured with peripheral arterial tonometry (PAT) signals, implies that adult patients with CF who have no cardiovascular (CV) risk factors have a significantly higher chance of endothelial dysfunction compared to healthy people. Endothelial dysfunction in such patients could be explained by two mechanisms. First, a direct result of the CFTR gene polymorphism. Second, due to systemic inflammation and oxidative stress associated with CF. These two mechanisms may coexist and potentiate each other [40].

CFTR protein channel is found in smooth muscle cells [41, 42] and vascular endothelium [43]. Alteration in CFTR compromises control of concentrations of Ca+2, which in turn decreases the activity of endothelial nitric oxide (NO) synthase resulting in reduced NO bioavailability. The dysfunctional CFTR also disrupts the endothelial barrier and stimulates the production of Interleukin 8 (IL-8), a powerful chemotactic agent, allowing leukocyte infiltration and inflammation. In short, decreased endothelial integrity along with attraction and infiltration of leukocytes may play an important part in determining endothelial dysfunction in patients with CF [40].

Cystic fibrosis is characterized by an increase in systemic inflammation and oxidative stress [41, 44] and both can further decrease NO bioavailability. Patients with CF show higher systemic inflammation with the presence of increased c-reactive protein (CRP) compared to healthy controls [45]. The elevated CRP in these patients is a result of recurrent chronic respiratory tract infections that are accompanied by a local and systemic inflammatory-immune response. Systemic inflammation affects endothelial function, resulting in arterial stiffness [46]. Besides that, the release of microvesicles from endothelial cells of patients with CF does not seem to stimulate cell proliferation adequately [47].

Limitations

This review paper has multiple limitations. Studies older than 25 years (1996-2021) or papers written in any other language than English were not included. Not enough studies have been carried out to explain the direct effect of CFTR gene mutation on the human heart.

Results

Totally, 12 articles were included in this study: Eight case-control, three prospective cohorts, and one case series. The characteristics of each study are outlined in Table 1.

**Table 1:** Literature review summary CF=Cystic fibrosis, LVEF=Left ventricular ejection fraction, LVF=Left ventricular function

No.	Author/year	Study Type	Number of Patients with CF	Results		
				RightVentricle	Left Ventricle	Blood vessel
1	Eising, J. B., et al. 2018 [15]	Case-Control	33 (Children)	Right ventricular dysfunction	Left ventricular dysfunction	Arterial stiffness
2	Sellers, Z. M., et al., 2015 [19]	Prospective Cohort	8 (Age >=18 years)	Normal right ventricular function	Left ventricular systolic & diastolic dysfunction	
3	Zebrak, J., et al. 2000 [20]	Case Series	n=18 (Children)	Myocardial fibrosis and necrosis		
4	Labombarda, F., et al., 2011 [23]	Prospective Cohort	42 (Age >=16)		Subclinical changes in LVF	
5	Koelling, T. M., et al., 2002 [24]	Case-Control	40	Right ventricular dysfunction	Left ventricular diastolic dysfunction	
6	Giacchi, V., et al., 2015 [25]	Case-Control	25 (Children & Adolescents), 30 (Adults)	Right ventricular systolic dysfunction	Decreased LVEF in adult patients	
7	Sciatti, E., et al. 2019 [33]	Case-Control	22	Right ventricular subclinical systolic-diastolic dysfunction	No left ventricular dysfunction	
8	Bano-Rodrigo, A., et al., 2012 [34]	Case-Control	37 (Adolescents)	abnormalities in right ventricular anatomy, systolic & diastolic function		
9	Ozcelic, N., et al., 2013 [35]	Case-Control	18 (Children/Adolescents)	Right ventricular dysfunction		
10	Florea, V., et al., 2000 [39]	Case-Control	103 (Adults)	Right ventricular systolic & diastolic impairment	No left ventricular anomalies	
11	Vizzardi, E., et al., 2019 [40]	Case-Control	22 (Adults)			Endothelial dysfunction
12	Poore, S., et al., 2013 [45]	Prospective Cohort	15 (Adults)			Endothelial dysfunction

## Conclusions

The review of these studies concludes that the cardiovascular system is directly or indirectly involved in CF. Cardiac dysfunction in CF is not only a complication of pulmonary hypertension caused by the destruction of lung parenchyma but also additional factors, such as CFTR gene polymorphism, hypoxia-induced myocardial damage, systemic inflammation, diabetic cardiomyopathy, and deficiency of some essential trophic factors. However, further studies are required for a better understanding of the mechanism and potential treatment options.

The focus on the cardiovascular system concerning CF has led to the emergence of numerous questions. Can the early detection of heart disease improve the survival rate of patients diagnosed with this disorder? If so, what are the best screening tests and when should they be conducted? Do certain mutations in the CFTR gene have a greater impact on the cardiovascular system? Irrespective of the answers, the research on these questions could help deepen the understanding of cardiac function in CF and lead to better clinical outcomes.

## References

[1] Davis PB (2006). Cystic fibrosis since 1938. Am J Respir Crit Care Med.

[2] Bethesda CFF 6931 AR 2nd floor, 800-344-4823 M 20814301-951-4422 (2016). About Cystic Fibrosis | CF Foundation. https://www.cff.org/What-is-CF/About-Cystic-Fibrosis/.

[3] Warth JD, Collier ML, Hart P (1996). CFTR chloride channels in human and simian heart. Cardiovasc Res.

[4] Sellers ZM, Kovacs A, Weinheimer CJ, Best PM (2013). Left ventricular and aortic dysfunction in cystic fibrosis mice. J Cyst Fibros.

[5] Sellers ZM, De Arcangelis V, Xiang Y, Best PM (2010). Cardiomyocytes with disrupted CFTR function require CaMKII and Ca(2+)-activated Cl(-) channel activity to maintain contraction rate. J Physiol.

[6] Jiang K, Jiao S, Vitko M, Darrah R, Flask CA, Hodges CA, Yu X (2016). The impact of cystic fibrosis transmembrane regulator disruption on cardiac function and stress response. J Cyst Fibros.

[7] Page MJ, McKenzie JE, Bossuyt PM (2021). The PRISMA 2020 statement: an updated guideline for reporting systematic reviews. BMJ.

[8] McKone EF, Emerson SS, Edwards KL, Aitken ML (2004). Effect of genotype on phenotype and mortality in cystic fibrosis: a retrospective cohort study. Lancet.

[9] Nagel G, Hwang TC, Nastiuk KL, Nairn AC, Gadsby DC (1992). The protein kinase A-regulated cardiac Cl- channel resembles the cystic fibrosis transmembrane conductance regulator. Nature.

[10] Gao Z, Sun HY, Lau CP, Chin-Wan Fung P, Li GR (2007). Evidence for cystic fibrosis transmembrane conductance regulator chloride current in swine ventricular myocytes. J Mol Cell Cardiol.

[11] Ling H, Zhang T, Pereira L (2009). Requirement for Ca2+/calmodulin-dependent kinase II in the transition from pressure overload-induced cardiac hypertrophy to heart failure in mice. J Clin Invest.

[12] Zhang T, Johnson EN, Gu Y (2002). The cardiac-specific nuclear delta(B) isoform of Ca2+/calmodulin-dependent protein kinase II induces hypertrophy and dilated cardiomyopathy associated with increased protein phosphatase 2A activity. J Biol Chem.

[13] Tilly BC, Bezstarosti K, Boomaars WE, Marino CR, Lamers JM, de Jonge HR (1996). Expression and regulation of chloride channels in neonatal rat cardiomyocytes. Mol Cell Biochem.

[14] Lader AS, Wang Y, Jackson GR Jr, Borkan SC, Cantiello HF (2000). cAMP-activated anion conductance is associated with expression of CFTR in neonatal mouse cardiac myocytes. Am J Physiol Cell Physiol.

[15] Eising JB, van der Ent CK, Teske AJ, Vanderschuren MM, Uiterwaal CS, Meijboom FJ (2018). Young patients with cystic fibrosis demonstrate subtle alterations of the cardiovascular system. J Cyst Fibros.

[16] Hume JR, Horowitz B (1995). A plethora of cardiac chloride conductances: molecular diversity or a related gene family. J Cardiovasc Electrophysiol.

[17] Yajima T, Nagashima H, Tsutsumi-Sakai R (1997). Functional activity of the CFTR Cl- channel in human myocardium. Heart Vessels.

[18] Chiba-Falek O, Parad RB, Kerem E, Kerem B (1999). Variable levels of normal RNA in different fetal organs carrying a cystic fibrosis transmembrane conductance regulator splicing mutation. Am J Respir Crit Care Med.

[19] Sellers ZM, McGlocklin L, Brasch A (2015). Strain rate echocardiography uncovers subclinical left ventricular dysfunction in cystic fibrosis. J Cyst Fibros.

[20] Zebrak J, Skuza B, Pogorzelski A (2000). Partial CFTR genotyping and characterisation of cystic fibrosis patients with myocardial fibrosis and necrosis. Clin Genet.

[21] Urheim S, Edvardsen T, Torp H, Angelsen B, Smiseth OA (2000). Myocardial strain by Doppler echocardiography: validation of a new method to quantify regional myocardial function. Circulation.

[22] Edvardsen T, Gerber BL, Garot J, Bluemke DA, Lima JA, Smiseth OA (2002). Quantitative assessment of intrinsic regional myocardial deformation by Doppler strain rate echocardiography in humans: validation against three-dimensional tagged magnetic resonance imaging. Circulation.

[23] Labombarda F, Pellissier A, Ellafi M (2011). Myocardial strain assessment in cystic fibrosis. J Am Soc Echocardiogr.

[24] Koelling TM, Dec GW, Ginns LC, Semigran MJ (2003). Left ventricular diastolic function in patients with advanced cystic fibrosis. Chest.

[25] Giacchi V, Rotolo N, Amato B (2015). Heart involvement in children and adults with cystic fibrosis: correlation with pulmonary indexes and inflammation markers. Heart Lung Circ.

[26] Brilla CG, Rupp H, Funck R, Maisch B (1995). The renin-angiotensin-aldosterone system and myocardial collagen matrix remodelling in congestive heart failure. Eur Heart J.

[27] Zimmermann A, Stocker F, Jöhr M, Torriani R, Chassot J, Weber JW (1982). Cardiomyopathy in cystic fibrosis: lymphoedema of the heart with focal myocardial fibrosis. Helv Paediatr Acta.

[28] Wiebicke W, Artlich A, Gerling I (1993). Myocardial fibrosis - a rare complication in patients with cystic fibrosis. Eur J Pediatr.

[29] Barnes GL, Gwynne JF, Watt JM (1970). Myocardial fibrosis in cystic fibrosis of the pancreas. J Paediatr Child Health.

[30] VanDevanter DR, Kahle JS, O'Sullivan AK, Sikirica S, Hodgkins PS (2016). Cystic fibrosis in young children: a review of disease manifestation, progression, and response to early treatment. J Cyst Fibros.

[31] Herrera-Garza EH, Stetson SJ, Cubillos-Garzon A, Vooletich MT, Farmer JA, Torre-Amione G (1999). Tumor necrosis factor-alpha: a mediator of disease progression in the failing human heart. Chest.

[32] Christie JD, Edwards LB, Aurora P (2008). Registry of the International Society for Heart and Lung Transplantation: twenty-fifth official adult lung and heart/lung transplantation report - 2008. J Heart Lung Transplant.

[33] Sciatti E, Vizzardi E, Bonadei I (2019). Focus on echocardiographic right ventricular strain analysis in cystic fibrosis adults without cardiovascular risk factors: a case-control study. Intern Emerg Med.

[34] Baño-Rodrigo A, Salcedo-Posadas A, Villa-Asensi JR, Tamariz-Martel A, Lopez-Neyra A, Blanco-Iglesias E (2012). Right ventricular dysfunction in adolescents with mild cystic fibrosis. J Cyst Fibros.

[35] Ozcelik N, Shell R, Holtzlander M, Cua C (2013). Decreased right ventricular function in healthy pediatric cystic fibrosis patients versus non-cystic fibrosis patients. Pediatr Cardiol.

[36] Ionescu AA, Ionescu AA, Payne N, Obieta-Fresnedo I, Fraser AG, Shale DJ (2001). Subclinical right ventricular dysfunction in cystic fibrosis. A study using tissue Doppler echocardiography. Am J Respir Crit Care Med.

[37] Labombarda F, Saloux E, Brouard J, Bergot E, Milliez P (2016). Heart involvement in cystic fibrosis: a specific cystic fibrosis-related myocardial changes?. Respir Med.

[38] Henno P, Maurey C, Danel C (2009). Pulmonary vascular dysfunction in end-stage cystic fibrosis: role of NF-kappaB and endothelin-1. Eur Respir J.

[39] Florea VG, Florea ND, Sharma R, Coats AJ, Gibson DG, Hodson ME, Henein MY (2000). Right ventricular dysfunction in adult severe cystic fibrosis. Chest.

[40] Vizzardi E, Sciatti E, Bonadei I (2019). Macro- and microvascular functions in cystic fibrosis adults without cardiovascular risk factors: a case-control study. Monaldi Arch Chest Dis.

[41] Robert R, Thoreau V, Norez C (2004). Regulation of the cystic fibrosis transmembrane conductance regulator channel by beta-adrenergic agonists and vasoactive intestinal peptide in rat smooth muscle cells and its role in vasorelaxation. J Biol Chem.

[42] Michoud MC, Robert R, Hassan M (2009). Role of the cystic fibrosis transmembrane conductance channel in human airway smooth muscle. Am J Respir Cell Mol Biol.

[43] Tousson A, Van Tine BA, Naren AP, Shaw GM, Schwiebert LM (1998). Characterization of CFTR expression and chloride channel activity in human endothelia. Am J Physiol.

[44] Rodriguez-Miguelez P, Thomas J, Seigler N, Crandall R, McKie KT, Forseen C, Harris RA (2016). Evidence of microvascular dysfunction in patients with cystic fibrosis. Am J Physiol Heart Circ Physiol.

[45] Poore S, Berry B, Eidson D, McKie KT, Harris RA (2013). Evidence of vascular endothelial dysfunction in young patients with cystic fibrosis. Chest.

[46] Halcox JP, Schenke WH, Zalos G (2002). Prognostic value of coronary vascular endothelial dysfunction. Circulation.

[47] Totani L, Plebani R, Piccoli A (2017). Mechanisms of endothelial cell dysfunction in cystic fibrosis. Biochim Biophys Acta Mol Basis Dis.

